# A Scoping Review Investigating Relationships between Depression, Anxiety, and the PrEP Care Continuum in the United States

**DOI:** 10.3390/ijerph182111431

**Published:** 2021-10-30

**Authors:** Sarah J. Miller, Sayward E. Harrison, Kamla Sanasi-Bhola

**Affiliations:** 1 Department of Psychology, College of Arts and Sciences, University of South Carolina, Columbia, SC 29208, USA; sjm8@email.sc.edu; 2 South Carolina Smart State Center for Healthcare Quality, Arnold School of Public Health, University of South Carolina, Columbia, SC 29208, USA; 3 Department of Internal Medicine, School of Medicine, University of South Carolina, Columbia, SC 29203, USA; Kamla.Sanasi@uscmed.sc.edu

**Keywords:** men who have sex with men, transgender women, HIV prevention, pre-exposure prophylaxis, anxiety, depression, mental health

## Abstract

Men who have sex with men and transgender women in the United States are at increased risk for HIV and may benefit from pre-exposure prophylaxis (PrEP), a once-a-day pill to prevent HIV. Due to stigma and discrimination, sexual and gender minority (SGM) populations are also at risk for depression and anxiety. This scoping review sought to identify literature addressing relationships between the PrEP care continuum, depression, and anxiety among SGM individuals and others at high risk for HIV. We conducted a systematic review of four databases (i.e., PubMed, PsycInfo, Web of Science, Google Scholar) and identified 692 unique articles that were screened for inclusion criteria, with 51 articles meeting the final inclusion criteria. Data were extracted for key study criteria (e.g., geographic location, participant demographics, study design, main findings). Results suggest that while depression and anxiety are not associated with PrEP awareness or willingness to use, they can be barriers to seeking care and to PrEP adherence. However, empirical studies show that taking PrEP is associated with reductions in anxiety. Findings suggest the need to implement mental health screenings in PrEP clinical care. In addition, addressing systemic and structural issues that contribute to mental health disorders, as well as PrEP-related barriers, is critical.

## 1. Introduction

Within the United States (US), ~1.2 million individuals were living with HIV in 2019 [1]. In that year alone, 36,801 individuals in the US and dependent areas were newly diagnosed with HIV [1]. Although the annual number of new HIV diagnoses has decreased since 2015 [1], continued efforts are needed to scale up prevention efforts to meet current federal goals to end the HIV epidemic [2]. The Center for Disease Control and Prevention (CDC) has identified gay, bisexual, and other men who have sex with men (MSM) and transgender persons as key populations for HIV prevention. Indeed, MSM accounted for 65% of new diagnoses in 2019, while MSM who also inject drugs accounted for an additional 4% [3]. Transgender persons accounted for 2% of the new HIV diagnoses in 2019, with transgender women (TGW) accounting for the vast majority (93%) of these [3]. This is likely an underestimate of prevalence because gender is sometimes reported incorrectly or is unavailable, leading to a reliance on sex rather than gender in reporting [4]. Approximately 14% of TGW are living with HIV, with greater rates amongst Black and Hispanic populations [5].

Pre-exposure prophylaxis (PrEP) is a once-a-day medication that, when taken regularly, reduces an individual’s risk of acquiring HIV by sex by 99% and by injection drug use by 74–84% [6,7,8]. Since initial approval by the US Food and Drug Administration (FDA) in 2012 under the brand name Truvada^®^, PrEP has also become available as a second formulation under the brand name Descovy^®^ and in a generic form under the brand name Truvada^®^ [6,9]. In addition, data from a clinical trial of a long-acting injectable (LAI-PrEP) have been published, though it has not received FDA approval as of September 2021 [10,11]. According to CDC guidelines, MSM who have a sexual partner living with HIV, have a recent bacterial sexually transmitted infection (STI), have multiple sex partners, or have a history of inconsistent or no condom use are potential candidates for PrEP [12]. These same PrEP indicators, as well as engagement in commercial sex work, apply for heterosexual men and women living in areas with high HIV prevalence [12]. Finally, for people who inject drugs (PWID), having an injecting partner living with HIV or sharing injection equipment is an indicator for PrEP [12].

The PrEP care continuum according to Nunn et al.’s conceptualization includes nine steps which can be used to assess PrEP programs. These steps fall into three general categories: (1) PrEP awareness, (2) PrEP uptake, and (3) PrEP adherence and retention [13]. PrEP awareness includes three steps: identifying individuals at high risk for HIV, increasing risk awareness, and enhancing PrEP awareness among these individuals. PrEP uptake includes four steps: facilitating PrEP access, linking to PrEP care, prescribing PrEP, and initiating PrEP. Finally, PrEP adherence and retention includes two steps: adhering to PrEP and retaining individuals in PrEP care [13].

Despite a strong safety profile and high efficacy for preventing HIV, uptake of PrEP has been slow and uneven in the US [14,15]. In addition, among those who do initiate PrEP, significant racial and ethnic disparities exist, with African American and Hispanic individuals being much less likely to use PrEP than White individuals, despite their higher HIV risk [16]. Multiple individual-level barriers to PrEP initiation and adherence have been identified, including lack of PrEP awareness and knowledge, concerns about side effects, lack of affirming healthcare providers, health concerns, concerns about PrEP’s potential role in future HIV drug resistence, and concerns about affordability [17,18,19]. Another important potential individual-level barrier to PrEP uptake and retention is mental health.

Sexual and gender minority (SGM) individuals—including MSM and TGW—are at increased risk for mental health disorders, such as depression and anxiety, due to experiences of stigma, prejudice, and discrimination [20]. The lifetime prevalence of depression within the general US population is 20% [21], compared with 26% for gay and bisexual men [22] and 51% for TGW [23]. Likewise, the lifetime prevalence of generalized anxiety disorder within the general US population is 6% [24], while it is 10% for gay and bisexual men [22] and 40% for TGW [23]. Given the prevalence of anxiety and depression in these groups, the World Health Organization (WHO) recommends screening for depression and other mental health conditions among those who test positive for HIV [25]. Recent calls have echoed this recommendation, suggesting that mental health services ought to be integrated into HIV prevention efforts as well [26,27]. In addition, some clinics, such as those funded by the Ryan White Program, currently use screening tools as a part of PrEP prescription.

Given the high prevalence of depression and anxiety among SGM and other populations at high risk for HIV, it is important to explore potential relationships between mental health disorders and the PrEP care continuum [13]. However, it is unclear to what extent research has been conducted on this topic. Thus, the present scoping review seeks to (1) identify existing literature examining potential bidirectional relationships between the PrEP care continuum and depression and anxiety, and (2) identify gaps in the existing literature and areas for future research.

## 2. Materials and Methods

We followed Arksey and O’Malley’s guidelines [28] for conducting a scoping review, adopting suggestions by Levac et al. [29] and Peters et al. [30] when feasible. The protocol was developed *a priori* but not pre-registered. It is available upon request from the corresponding author. We followed five steps in conducting the scoping review: (1) identifying the research question; (2) identifying relevant studies; (3) study selection; (4) charting the data; and (5) collating, summarizing, and reporting the results [28].

Two of the authors (SM and SH) collaboratively developed the search strategy and inclusion criteria. Key terms related to PrEP, anxiety, depression, and mental health were used to search PubMed, PsycInfo, Web of Science, and Google Scholar. Detailed search criteria are shown in Table 1. The final search was conducted on 27 August 2021. All results from PubMed, PsycInfo, and Web of Science were screened, and the first 300 results from Google Scholar were screened, as recommended for searches aimed at academic literature [31]. The following inclusion criteria were used: (1) full text article written in English, (2) conducted in US, (3) published in a peer-reviewed journal, (4) published between January 2012 and July 2021, (5) described original qualitative or quantitative research, (6) included participants on PrEP or PrEP candidates (i.e., individuals from groups at high risk for HIV including MSM, transgender individuals, and PWID), and (7) included measures of depression and/or anxiety (i.e., symptoms or clinical diagnoses) as key study variables (e.g., independent variable, dependent variable, mediator, moderator, inclusion criteria, or qualitative focus) in relation to the PrEP care continuum step(s). The review was limited to articles written in English due to study team constraints. It was limited to studies conducted in the United States because of the varying nature of HIV epidemics and presentation of mental health disorders across geographic and cultural settings. Finally, 2012 was selected as the beginning year for inclusion as that was the year of PrEP’s initial approval in the US [6].

First, duplicate results were removed. To screen articles, the first author worked collaboratively with a trained research assistant. The primary coder screened articles based on title and abstract, removing studies that clearly did not meet inclusion criteria. The secondary screener reviewed initial screening decisions in an unblinded fashion. The two screeners met to discuss any discrepancies in screening and come to a resolution. Next, the primary coder screened articles based on full text. The secondary coder reviewed all excluded articles to ensure accuracy. For all included articles, references were searched based on title to identify studies that were not found through the database searches. In addition, articles that cited the included studies, as identified through the “cited by” function in Google Scholar, were also searched based on title for possible inclusion.

To chart the data, the following were extracted from each article by the first author: author(s), year of publication, study location(s), trial name when applicable, study design, sample demographics, sampling strategy, study aims, mental health disorder(s) of focus, tools/measures used to assess mental health, tools/measures used to assess PrEP outcomes, main findings, and study limitations. The extracted variables are included in tables, with the exception of trial name, sampling strategy, and study limitations. These data are included in the narrative description of studies, when applicable.

To synthesize the findings, reviewed articles were then grouped by mental health disorder (i.e., depression and/or anxiety) and PrEP care continuum step (i.e., PrEP awareness, PrEP uptake, and/or PrEP adherence and retention). The authors examined and discussed the findings for each mental health disorder at each step of the PrEP care continuum.

## 3. Results

**Figure 1:**
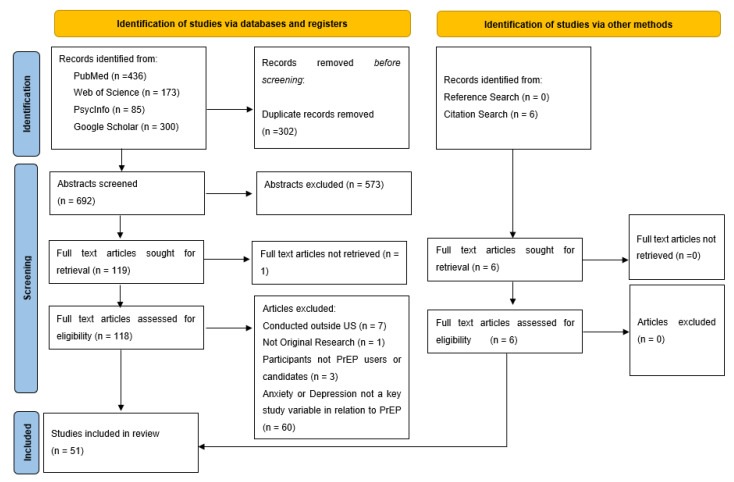
PRISMA flow chart [32].

Our search yielded 994 initial articles (436 PubMed, 85 PsycInfo, 173 Web of Science, 300 Google Scholar). A total of 302 duplicates were identified and removed, yielding 692 unique articles. After screening all titles/abstracts, an additional 573 were removed, resulting in 119 articles that were included for full text screening. One article was not available for full text review, and its corresponding author did not reply to a request to share a full text version. Thus, 118 full text articles were screened. Of these, 45 were identified for inclusion. Screening references and citations of these included articles yielded six additional articles that met the inclusion criteria. Therefore, a total of 51 articles are included in this scoping review. See Figure 1 for a flow chart detailing this process.

## 4. Depression

### 4.1. PrEP Awareness

Fifteen articles were identified that examined depression and PrEP awareness and/or willingness to use PrEP (see Table 2). The majority of these studies found that depressive symptoms were not correlated with PrEP awareness or willingness to use PrEP.

Five studies that recruited MSM from major US cities found that a positive screening for depressive symptoms was not associated with PrEP awareness [33,34,35,36,37] or reported willingness to use [36]. This was shown through cross-sectional surveys of 1274 Black MSM across the US [33]; 374 MSM in Nashville, Tennessee (TN) and Buffalo, New York (NY) [36]; and 293 MSM in Atlanta, Georgia (GA) [37]; as well as a serial cross-sectional survey of 2398 Black MSM [34]; and a repeated cross-sectional survey with 398 MSM at time 1 and 558 MSM at time 2 [35]. Another cross-sectional study of 234 individuals who were dependent on opioids similarly found no relationship between depressive symptoms and PrEP awareness [38].

Three studies found no relationship between depressive symptoms and PrEP awareness and interest in samples of women. A cross-sectional study with 232 female sex workers found that depressive symptoms were not associated with PrEP awareness or interest [39]. Among 190 women in New York City, NY, another cross-sectional study found that depression was not associated with PrEP interest [40]. Finally, in a cross-sectional study of 190 predominantly African American women, depression was not related to willingness to use PrEP [41].

Two cross-sectional studies examined depression as a part of syndemic conditions, finding no difference in PrEP interest between those with and without syndemic conditions. One study found that among 271 women at risk for HIV, having syndemic factors, including depression, alcohol consumption, illicit drug use, or transactional sex was not associated with lower likelihood of PrEP interest or intentions to use when controlling for sociodemographic variables [47]. Similarly, a study conducted with 151 Latino gay, bisexual, and other MSM in San Diego, California (CA) found that syndemic conditions, including depression, binge drinking, marijuana use, illicit polysubstance use, and childhood sexual abuse, were not associated with PrEP awareness or willingness to use [33].

Cross-sectional studies involving people who use drugs yielded differential results for the daily PrEP pill compared with LAI-PrEP. In a study conducted in Washington DC, among 304 predominantly Black PWID, those who screened positive for depressive symptoms were more likely to be willing to use PrEP compared with those who did not screen positive for depressive symptoms [39]. Likewise, in a sample of 400 individuals enrolled in a methadone program in New Haven, Connecticut (CT), those with moderate to severe depression were more likely to be willing to use PrEP compared with those with no to mild depression [46]. However, in a study involving 234 individuals enrolled in a methadone program in New Haven, CT, depression was not correlated with willingness to use LAI-PrEP [45].

A final study examined willingness to use PrEP amongst a predominantly Black study population of 855 participants from the US. Within the entire sample, depressive symptoms were associated with greater willingness to use PrEP. However, when the sample was limited to those who had clear PrEP indications according to CDC guidelines, this relationship disappeared [44].

### 4.2. PrEP Uptake

A total of 23 articles were identified that evaluated depression and outcomes of PrEP access, uptake, and/or actual use (see Table 3).

*PrEP Access.* Three qualitative studies were identified that found depression to be a barrier to PrEP access. A study conducted in southern CA with 30 TGW found that mental health concerns were identified as barriers to PrEP care [48]. Similarly, another study conducted in southern CA with 37 individuals, the majority of whom were transgender, identified that mental disorder symptoms made PrEP access difficult [49]. Similarly, in a study of 25 PrEP users in San Francisco, CA, one participant identified depression-related and HIV-related stigma as a barrier to seeking care [50].

*Linking to PrEP Care.* Three studies were identified which examined components of linking individuals to PrEP care. First, in a cross-sectional study conducted in CT with 125 women involved in the criminal justice system, depressive symptoms were not associated with PrEP eligibility as measured by CDC criteria [51]. Second, in a retrospective chart review of 2995 PrEP candidates in southern California, depression according to diagnostic codes in medical charts was not associated with PrEP referral [52]. Finally, in a study with 52 women in New York City, depression was not correlated with acceptance of an appointment to discuss PrEP [40].

*PrEP Initiation.* Seventeen additional articles detailed findings on depression and PrEP initiation and/or use. Four of these studies examined PrEP initiation based on depressive symptoms and found no relationship. In a longitudinal analysis of 154 Black MSM in Atlanta, GA, depression was not associated with a delay between receiving a PrEP prescription and initiating PrEP [53]. Similarly, in a prospective observational cohort study with 298 non-Hispanic Black cisgender MSM in Atlanta, GA, depressive symptoms were not associated with PrEP uptake as defined by the date of taking the first PrEP dose after prescription [54]. Likewise, in two studies that examined a sample of 226 predominantly Black MSM in Los Angeles, CA, Washington DC and Chapel Hill, North Carolina (NC), there was no relationship between depressive symptoms and PrEP initiation as defined as self-reported first date of taking PrEP [55,56].

Thirteen studies examined differences in depressive symptoms between PrEP users and non-users in cross-sectional analyses with somewhat inconsistent findings. Three studies with MSM found no association between depression and PrEP use [34,35,36]. This was shown in a serial cross-sectional survey design with 2398 Black MSM [34], a repeated cross-sectional survey design with 398 MSM at time 1 and 558 MSM at time 2 [35], and a cross-sectional design with 374 MSM [36]. Similarly, in a cross-sectional study of 6283 predominantly cisgender men that did not report sexual identity or behaviors, depressive symptoms were not correlated with PrEP use [59]. In a cross-sectional study of 247 predominantly cisgender males from Philadelphia, Pennsylvania (PA), there was no relationship between depressive symptoms and self-reported PrEP use [58]. Likewise, in a cross-sectional study conducted with 234 individuals dependent on opioids, moderate to severe depression was not correlated with PrEP use [38]. However, some studies have found a relationship between depression and PrEP use. In a cross-sectional survey of 2406 MSM in the US, PrEP users reported lower levels of depression than non-PrEP users. However, in regression analyses, use of PrEP did not predict depression [62]. In a cross-sectional survey of 4184 Black MSM and TGW, higher depressive symptoms were associated with a greater likelihood of being a current PrEP user compared with non-PrEP users [61]. Likewise, another cross-sectional study of 1274 Black MSM and TGW showed via bivariate analyses that depressive symptoms were positively associated with PrEP use [33]. However, this association disappeared when depressive symptoms were included as a part of multivariable analyses [33].

In a cross-sectional survey of 1411 Black MSM, PrEP users and non-users did not differ in depressive symptoms [60]. However, when considered as a part of syndemic psychosocial conditions, including substance use, intimate partner violence, and depression, greater syndemic conditions were associated with a greater likelihood of PrEP use [60]. Similarly, in another cross-sectional survey of 293 Black MSM, depression alone was not associated with PrEP use, while a syndemic of two or more symptoms including depression, alcohol consumption, illicit drug use, and transactional sex was associated with a lower likelihood of PrEP use [37]. In contrast, a cross-sectional study conducted with 151 Latino MSM in San Diego, CA found that a psychosocial syndemic of depression, binge drinking, marijuana use, illicit polysubstance use, and/or childhood sexual abuse was not associated with PrEP use [42].

Finally, a cross-sectional survey of 9697 PrEP users found that those who used PrEP in an event-driven non-daily fashion were more likely to have depressive symptoms compared with those who used PrEP daily [57].

### 4.3. PrEP Adherence and Retention

A total of 17 articles were included in the review that evaluated depression and outcomes of PrEP adherence and efficacy (see Table 4). Findings related to depressive symptoms and PrEP adherence and discontinuation were mixed, with some studies identifying no association, while others showed poorer adherence and higher rates of PrEP discontinuation.

*Adherence.* A total of 12 studies examined associations between depression and PrEP adherence. Six of these studies found that depressive symptoms were unrelated to adherence. This lack of relationship was shown in a cross-sectional survey of 31 cisgender males and TGW in Philadelphia, PA [63]; a longitudinal analysis of 280 predominantly male PrEP users in Miami, Florida (FL), San Francisco, CA, and Washington DC [64]; a longitudinal analysis of 557 PrEP users from Miami, FL, San Francisco, CA, and Washington DC [65]; a non-randomized open-label PrEP study of 226 MSM in Los Angeles, CA [55]; a randomized control trial with 394 MSM and TGW in southern CA [66]; and a randomized control trial conducted in New York City among 176 MSM [67].

In contrast, three studies found that depression was associated with poor adherence. In a randomized control trial of 204 Black MSM and TGW in Harlem, NY, those who reported depression at baseline were less likely to be adherent to PrEP [68]. As a part of the TAPIR longitudinal study with 181 MSM in southern CA, those with the poorest adherence were more likely to have depression compared with those with the best adherence [69]. Finally, one cross-sectional study with 151 Latino MSM examined a psychosocial syndemic, including depression, binge drinking, marijuana use, illicit polysubstance use, and childhood sexual abuse, which was associated with lower adherence. However, the analyses did not differentiate between those with depression and those with other mental health conditions [42].

Three qualitative studies found that depression hindered PrEP adherence. In a study conducted in San Francisco, CA, one participant identified that depression was a barrier to PrEP adherence, noting the link between depression and substance use as a particular challenge. [70]. Another study conducted in San Francisco with 25 PrEP users found that mental health problems were associated with adherence difficulties, although participants did not indicate what specific symptoms they experienced [50]. Finally, in a study of 30 TGW in southern CA, participants indicated that mental health conditions, including depression, decreased their motivation to take PrEP. PrEP adherence was a lower priority compared to mental health symptoms. Of note, some participants indicated that taking their PrEP on schedule was a way for them to positively cope with mental health symptoms [48].

*Retention.* Five studies examined PrEP discontinuation, four of which found no relationship between depression and PrEP discontinuation. In a prospective observational cohort study of 298 non-Hispanic Black cisgender MSM in Atlanta, GA, baseline moderate to severe depressive symptoms were not associated with PrEP discontinuation [54]. Likewise, a retrospective chart review of 696 PrEP users in Manhattan, NY found that depressive symptoms were not correlated with PrEP retention [73]. A retrospective chart review of 348 PREP users at San Francisco Department of Health primary care clinics found that a clinical diagnosis of depression noted in a patient’s chart was not associated with PrEP discontinuation [72]. Similarly, in a retrospective chart review of 2995 PrEP candidates in southern CA, diagnostic codes for depression were not associated with PrEP persistence as measured by medical or pharmacy records [52]. In contrast, in a retrospective chart review of 663 PrEP users at a community health center in Boston, Massachusetts (MA), those with multiple mental health medical problem lists or diagnostic codes were more likely to discontinue PrEP [71]. Importantly, this study examined anxiety, depression, substance use, PTSD, bipolar disorder, schizophrenia, and attention deficit disorder, and did not include analyses for individual disorders [71].

## 5. Anxiety

A total of 10 studies were identified that examined the impact of anxiety on various PrEP stages (see Table 5).

### 5.1. PrEP Awareness

Two studies were identified that examined anxiety and PrEP awareness, PrEP interest, and willingness to take PrEP. In a cross-sectional sample of 374 young, black MSM in Nashville, TN and Buffalo, NY, anxiety was not associated with PrEP awareness, willingness to use PrEP, or actual PrEP use [36]. Likewise, in a cross-sectional sample of 52 cisgender women and TGW in East Harlem and the Bronx, NY, anxiety was not associated with PrEP interest [40].

One study found a relationship between anxiety and perceptions of LAI-PrEP. Twenty-eight participants, predominantly MSM of color, were recruited from a study examining long-acting PrEP injections. They qualitatively reported anxiety related to needles and expected pain from the injection. However, this anxiety decreased across time, and was not directly related to actual felt pain [74].

### 5.2. PrEP Uptake

*PrEP Access.* One qualitative study using a focus groups of 37 predominantly transgender individuals in southern CA identified mental health concerns, including anxiety, to be a barrier to PrEP care access. Participants reported these mental health concerns making it difficult to access both PrEP care and other important health care services [49].

*Linking to PrEP Care.* One cross-sectional sample of 52 cisgender women and TGW in East Harlem and the Bronx, NY found that anxiety was not associated with acceptance of a PrEP appointment.

*PrEP Initiation.* One large national US cohort study, enrolling 6283 cisgender men and transgender individuals who have sex with men demonstrated that anxiety symptoms were not associated with self-reported individual prior or current use of PrEP [59].

### 5.3. PrEP Adherence and Retention

*Adherence.* Two studies found that anxiety is associated with poor PrEP adherence. In a cross-sectional survey of 31 MSM and TGW in Philadelphia, PA, greater anxiety symptoms were associated with poorer PrEP adherence, as measured through urine tenofovir concentration testing [63]. However, there was no association between anxious symptoms and adherence to medical visits [63]. Similarly, qualitative findings from a study involving 30 TGW in southern CA revealed reports that mental health conditions including anxiety decreased individuals’ motivation to take PrEP, making adherence difficult [48]. Importantly, however, some participants indicated that taking PrEP was a way in which they coped with mental health conditions [48].

*Retention.* Three retrospective chart reviews have examined anxiety and PrEP discontinuations with somewhat mixed findings. In one large study of 2995 PrEP candidates receiving care in southern CA, clinical mental health diagnoses including anxiety and depression were not associated with PrEP maintenance [52]. Likewise, a retrospective chart review of 348 PrEP patients receiving care in San Francisco, CA indicated that clinical diagnoses of anxiety were not associated with PrEP discontinuation [72]. In contrast, a study of 663 PrEP users in Boston, MA showed that those with multiple mental health disorders indicated on medical charts, including anxiety, depression, substance use, PTSD, dipolar disorder, schizophrenia, and attention deficit disorder, were more likely to discontinue PrEP [71]. However, analyses did not examine the effect of anxiety specifically on PrEP discontinuation.

### 5.4. Effects of PrEP Use on Anxiety

Twelve studies examined the effects of taking PrEP on anxiety; these are detailed in Table 6. Ten of the reviewed qualitative studies with samples sizes ranging from 10 to 89 suggest that positive perceptions of PrEP may be associated with reduced anxiety. The studies primarily examined perceptions of men who identified as gay, bisexual, or other MSM. However, one included men on PrEP without defining sexual identity or activity [75]. In addition, one study included cisgender women and TGW; however, the vast majority of the sample (93%) was cisgender men [76]. Participants reported a reduction in anxiety generally [77,78], sex-related anxiety [75], HIV-related anxiety [76,79,80,81,82], and anxiety related to partner dishonesty [82]. Participants described PrEP as providing “peace of mind” [75,78,82]. Participants also identified PrEP as a possible component of couple’s sexual agreements, in order to reduce sexual anxiety [83]. Moreover, a reduction in anxiety was associated with increased feelings of control [80], responsibility [76], and enhanced sexual wellbeing [75,79,80,81]. Indeed, in one study, participants identified reduced anxiety as a primary motivator for willingness to take PrEP [84].

Two quantitative studies yielded similar results [62,85]. In a large cross-sectional study of 2406 MSM, PrEP users reported lower levels of anxiety symptoms compared with non-PrEP-users [62]. Moreover, while a greater number of condomless anal sex partners was associated with heightened anxiety symptoms in those not on PrEP, this relationship was not present for those on PrEP, suggesting that PrEP use buffers the effect of number of condomless anal sex partners on anxiety symptoms [62]. In a large longitudinal analysis of 1071 cisgender MSM, sexual anxiety decreased for individuals after initiating PrEP [85].

## 6. Discussion

This scoping review examined current peer-reviewed US-based studies exploring associations among depression, anxiety, and the PrEP care continuum for SGM individuals and others at high risk for HIV. Findings indicate that depression and anxiety have distinct relationships with the PrEP care continuum. The majority of studies reviewed found that depression was not associated with PrEP awareness or willingness to use PrEP. In fact, most of those studies that did identify a relationship showed that depressive symptoms were associated with a *higher* likelihood of being willing to use PrEP to prevent HIV.

Although depressive symptoms have been identified as barriers to PrEP care [49,50], this review suggests that depressive symptoms alone do not stymie PrEP uptake [56,58]. Indeed, cross-sectional analyses have found depressive symptoms to be positively associated with PrEP use [33,61,62]. However, due to their cross-sectional nature, these studies cannot make causal or temporal inferences. In addition, two studies found that depression alone was not associated with PrEP use, although it was when depression was part of syndemic conditions [37,60]. The salience of syndemic risks and health conditions among SGM has been highlighted previously, with prior research showing that SGM individuals are disproportionately impacted by intersecting psychosocial risks (e.g., substance use, depression, trauma) that together create challenging dynamics for physical health [86,87,88]. Current findings echo previous calls for novel assessment and intervention strategies that target multiple co-occurring risks among SGM populations [86]. In other words, clinicians and PrEP providers should be careful to not consider depression in isolation when discussing/recommending PrEP—but rather, should pay close attention to a constellation of risks (e.g., depressive symptoms, substance use, social isolation) that, taken together, may create significant barriers to PrEP initiation and adherence.

The majority of the studies that examined depression and PrEP focused on the role of depression in PrEP adherence, with largely mixed findings. Although the majority of identified articles found no relationship between depression and adherence, several studies showed that depression was negatively associated with PrEP adherence [42,68,69]. One study examining data from the iPrEX open label extension, which is not included in the present scoping review due to its global nature and aggregated findings, found that moderate levels of depressive symptoms were associated with the highest level of adherence, compared with severe and mild levels of depressive symptoms. Individuals with severe symptoms of depression had the worst adherence of all groups [89]. Thus, it is possible that depressive symptoms have a non-linear relationship with adherence, which may explain previous mixed findings on the relationship between depression and adherence.

Compared with depression, there has been relatively less research on anxiety and the PrEP care continuum. Overall, the literature reviewed here suggest that anxiety has limited impact on PrEP awareness, willingness to use, or actual use among SGM individuals. However, anxiety symptoms were identified as a potential barrier to care in qualitative studies. In addition, while some studies found that anxiety was associated with poorer adherence, it was not associated with PrEP discontinuation. The relationship between anxiety and PrEP is likely a bidirectional one. A sizeable majority of the studies examining PrEP and anxiety examined the effect PrEP had on anxiety through qualitative methods, with participants indicating the ways in which PrEP brought them peace of mind. This peace of mind may provide a sense of sexual freedom, enabling individuals to have sex without HIV-related anxiety. This is related to the concept of risk compensation, which, when applied to PrEP, posits that use of PrEP would reduce an individual’s perceived risks, which enables them to engage in other sexual risk behaviors [90]. Although risk compensation has been used as an argument against PrEP prescription, this view has been critiqued [90]. However, risk compensation may explain the reductions in anxiety related to condomless anal sex for MSM taking PrEP compared with those not taking PrEP.

### 6.1. Current Gaps and Future Directions

A number of gaps in existing research on the role of depression and anxiety in the PrEP care continuum were identified through the current scoping review. First, more clarity is needed on the relationship between depression and PrEP use, including longitudinal studies that examine these relationships over time in order to assess potential bidirectional temporal changes. Second, measures used to examine depression in PrEP-related research should be expanded. As previously discussed, Mehrotra and colleagues uncovered a non-linear relationship between depression and adherence by employing a categorical measure [89]. The vast majority of the current research has used dichotomous categorizations for depression, which do not allow for this nuanced understanding. Therefore, future research should consider expanding measures used to evaluate depressive symptoms beyond brief screening measures and consider multi-categorical analyses in addition to traditional cut-score methods. For example, the Center for Epidemiologic Studies Depression (CESD) scale yields scores ranging from 0 to 60, with a traditional screening cut-score of 16 [91]. However, analyses with multiple categories (e.g., mild, moderate, severe depression) may reveal important differences. Finally, future research should examine depression and the PrEP care cascade for TGW specifically. Although many of the current studies included TGW, the samples of TGW tended to be so small as to prohibit both cross-comparison with MSM and within-group analyses. Because rates of depression and HIV are so high among TGW, studies that focus exclusively on this population are urgently needed.

In terms of anxiety and the PrEP care continuum, this review revealed that when compared to depression, there is a dearth of research on the impact of anxiety on the PrEP care continuum. In addition, most studies that were reviewed were based in large metropolitan areas on the East or West coast. This highlights a current geographic gap—SGM in the southern US and rural areas are disproportionately impacted by the HIV epidemic, and these areas are key targets in the new federal Ending the HIV Epidemic plan [92]. However, there is limited research on anxiety and PrEP emerging from these key geographic areas. For example, the two studies that were reviewed and found no relationship between anxiety and PrEP awareness as well as willingness to use PrEP were both conducted in NY [,36, 40]. Given different sociocultural environments, it is possible that geographic differences could affect the role of anxiety in PrEP perceptions or willingness to seek and use PrEP. The ‘Deep South’, in particular, has become the epicenter of the US HIV epidemic and now has higher HIV rates and greater HIV-associated morbidity and mortality than any other region [92,93]. Multiple drivers of these regional HIV disparities exist, including systemic racism, homophobia, and transphobia; socio-cultural factors (e.g., socio-religious norms, stigmatized sexuality); healthcare disparities; structural barriers (e.g., housing, transportation); and policy-related factors (e.g., lack of Medicaid expansion) [92,94]. Taken together, these may certainly have an effect on anxiety of SGM and their desires or attempts to access PrEP. Expanding the geographic and social contexts of this research is important across the care cascade. In addition, more research is needed to examine the effects of PrEP on anxiety within populations other than MSM. Similar to depression, future research is also needed to examine longitudinal changes in anxiety symptoms as a result of PrEP use.

Finally, researchers should consider multiple ways of operationalizing anxiety. Most of the studies identified used dichotomous classifications for anxiety or qualitative reports of anxiety. Although these are useful, this method of analysis does not allow for a distinction between mild, moderate, or severe anxiety symptoms. Only one identified study used this method [40]. It may be that symptom severity influences outcomes. Prior research on Chronic Hepatitis C treatment adherence has supported the application of the Yerkes–Dodson law related to anxiety and treatment adherence, such that moderate levels of anxiety were related to best adherence [95]. Similarly, it may be that a certain level of anxiety is adaptive, while high levels of anxiety are harmful in PrEP adherence. More research is needed to understand this complex relationship.

Given the importance of anxiety and depression on the PrEP care continuum, healthcare providers prescribing PrEP should be aware of mental health considerations and be ready to implement screening protocols and provide referrals for mental health services for those with symptoms of anxiety and depression [17]. Alternatively, a self-screening for depression and substance use can be implemented, as done at a hospital in Toronto, Canada, with this method found to be acceptable and feasible [96]. By including such screenings, practitioners can link patients to mental healthcare providers, thus meeting the physical and mental healthcare needs of individuals. In addition, adherence-related interventions are likely appropriate for those experiencing anxiety and depression. One such intervention is the “Life-Steps” approach, which uses cognitive-behavioral techniques and motivational interviewing to support creating dosing schedules and problem solving for potential adherence barriers [97,98]. Advances in mobile technology also allow for mHealth interventions, such as text reminders, which are effective in improving adherence [99]. Despite the potential challenges related to mental health disorders, some symptoms may also help to motivate PrEP uptake. For instance, in some studies, moderate depressive symptoms were associated with greater interest; similarly, a desire to reduce anxiety surrounding condomless sex can also be a motivator for PrEP uptake.

### 6.2. Limitations

This scoping review has several limitations. First, the scope of the review was limited to US-based research. Although this was purposeful due to the varying nature of the HIV epidemics and presentation of depression and anxiety across cultural and geographic settings, this limits the generalizability of the summarized findings. Important findings related to anxiety or depression and PrEP may have been overlooked due to this decision. Second, the review focused on studies that included those on PrEP or who were PrEP candidates. This eliminated studies that focused on the perceptions of healthcare and mental health providers, which could provide insights into whether an individual is likely to receive a prescription, a key component to the PrEP care continuum. Third, although narrative descriptions of studies included informal analyses of article quality (e.g., study design, sample size), no formal quality assessment was conducted. Finally, many of the studies reviewed utilized cross-sectional data, prohibiting an understanding of potential causal relationships between PrEP and mental health.

## 7. Conclusions

The high prevalence of depression, anxiety, and HIV among SGM populations makes it important to consider the relationship between these mental health disorders and the PrEP care continuum. The present scoping review reveals that while anxiety and depression may not have major impacts on PrEP awareness or PrEP willingness, they can be barriers to access to care and can impact an individual’s adherence to PrEP. Moreover, the relationship between PrEP and these mental health conditions appears to be bidirectional. In particular, PrEP use reduces feelings of anxiety. Practitioners should implement mental health screenings to ensure PrEP candidates and users with mental health conditions are linked to appropriate care.

## Figures and Tables

**Table 1:** Search Terms.

	PubMed	PsycInfo	Web of Science	Google Scholar
PrEP	(Pre-Exposure Prophylaxis[Mesh] OR “pre-exposure prophylaxis”[Title/Abstract] OR “preexposure prophylaxis”[Title/Abstract] OR “pre exposure prophylaxis” [Title/Abstract] OR PrEP [Title/Abstract] OR “HIV PrEP” [Title/Abstract] OR tenofovir [Title/Abstract] OR truvada [Title/Abstract] OR emtricitabine [Title/Abstract] OR descovy [Title/Abstract])	(DE(“Pre-Exposure Prophylaxis”) OR AB (“pre-exposure prophylaxis” OR “preexposure prophylaxis” OR “pre exposure prophylaxis” OR PrEP OR “HIV PrEP” OR tenofovir OR truvada OR emtricitabine OR descovy) OR TI (“pre-exposure prophylaxis” OR “preexposure prophylaxis” OR “pre exposure prophylaxis” OR PrEP OR “HIV PrEP” OR tenofovir OR truvada OR emtricitabine OR descovy))	(AB = (“pre-exposure prophylaxis” OR “preexposure prophylaxis” OR “pre exposure prophylaxis” OR PrEP OR “HIV PrEP” OR tenofovir OR truvada OR emtricitabine OR descovy) OR TI = (“pre-exposure prophylaxis” OR “preexposure prophylaxis” OR “pre exposure prophylaxis” OR PrEP OR “HIV PrEP” OR tenofovir OR truvada OR emtricitabine OR descovy))	(PrEP OR “pre-exposure prophylaxis” OR “pre exposure prophylaxis” OR “preexposure prophylaxis” OR tenofovir OR truvada OR descovy)
	AND	AND	AND	AND
Anxiety	“Anxiety”[Mesh] OR “Anxiety Disorders”[Mesh] OR anxiety[Title/Abstract] OR anxious[Title/Abstract]	DE(“Anxiety” OR “Anxiety Disorders” OR “Generalized Anxiety Disorder”) OR AB(anxiety OR anxious) OR TI (anxiety OR anxious)	AB = (“generalized anxiety disorder” OR “anxiety disorder” OR anxiety OR anxious) OR TI = (“generalized anxiety disorder” OR “anxiety disorder” OR anxiety OR anxious)	anxiety OR anxious
	OR	OR	OR	OR
Depression	(“Depression”[Mesh] OR “Depressive Disorder”[Mesh] OR “Depressive Disorder, Major”[Mesh] OR depress*[Title/Abstract])	(DE (“Major Depression” OR “Depression(Emotion)”) OR AB(depress *) OR TI(depress *)	(AB = (“major depression” OR “depressive disorder” OR “major depressive disorder” OR depress *) OR TI = (“major depression” OR “depressive disorder” OR “major depressive disorder” OR depress *)	depression OR depressive OR depress *
	OR	OR	OR	OR
Mental Health	(“Mental Health”[Mesh] OR “Mental Disorders”[Mesh] OR “mental health”[Title/Abstract] OR “mental disorder”[Title/Abstract])	(DE (“Mental Health” OR “Mental Disorders”) OR AB(“mental health” OR “mental disorder”) OR TI (“mental health” OR “mental disorder”))	(AB = (“mental health” OR “mental disorder”) OR TI = (“mental health” OR “mental disorder”))	(“mental health” OR “mental disorder”)
Limiters	From: 2012/1/1 English Article Type: Journal Article	Publication Year: 2012–2021 Source Types: Academic Journals	Language: (English) Document Types (Article) Timespan = 2012–2021	NA

Note: * indicates a truncated search technique yielding results for all terms which begin with the listed root.

**Table 2:** Depression and PrEP Awareness.

Authors & Year	Location(s)	Sample Demographics	Design	Depression Measure (Scoring)	Depression Related Findings
Blashill, Brady, Rooney et al. 2020 [42]	San Diego, CA	151 Latino MSM; mean age: 24.	Cross-sectional survey	PHQ-8 (10+ screened positive)	A psychosocial syndemic (depression, binge drinking, marijuana use, illicit polysubstance use, childhood sexual abuse) was not associated with PrEP awareness or willingness to use.
Blackstock, Platt, Golub et al. 2021 [40]	East Harlem, NY; Bronx, NY	52 women, 11.5% TGW; 34.6% non-Latina Black; 51.9% Latina/Hispanic, 42.3% LGB; mean age: 45.	Pilot test	PHQ-2 (dichotomous screening)	Depression was not associated with PrEP interest.
Bologna, Panesar-Aguilar, McCraney, and Cale 2020 [41]	United States	190 female individuals; 90% African American; mean age: 22.	Cross-sectional survey	PHQ-9 (continuous)	Depression was not related to willingness to use PrEP.
Eaton, Matthews, Driffin et al. 2017 [33]	Atlanta, GA; Detroit, MI; Houston, TX; Philadelphia, PA; Washington DC	1274 Black MSM and TGW; 96% Male; 76% gay/same gender loving; mean age: 30.	Cross-sectional survey	CESD-10 (10+ screened positive)	Depression not associated with PrEP awareness.
Friedman, Sang, Bukowski et al. 2019 [34]	Atlanta, GA; Detroit, MI; Houston, TX; Memphis, TN; Philadelphia, PA; Washington DC	2398 Black MSM, 82% MSM only; 3.4% Hispanic/Latino; mean age: 29.	Serial cross-sectional survey	CESD-10 (10+ screened positive)	Depressive symptoms was not associated with PrEP awareness.
Krakower, Mimiaga, Rosenberger et al. 2012 [35]	United States	398 MSM 2 months prior to iPrEX trial results publication; 82% White; 89% Gay; mean age: 40. 558 MSM 2 months following publication; 84% White; 83% gay; mean age: 39.	Repeated cross-sectional survey	CESD-10 (10+ screened positive)	Depression was not associated with PrEP awareness.
Kuo, Olsen, Patrick et al. 2016 [43]	Washington, DC	304 PWID; 69% male; 97.7% Black; 83% 50+ yrs.	Cross-sectional survey	CESD-10 (dichotomous screening)	Those who screened positive for depressive symptoms were more likely to be willing to use PrEP.
Liu, Brown, Przybyla et al. 2021 [36]	Nashville, TN; Buffalo, NY	374 MSM, 60.2% Black; 77% gay/homosexual; median age: 26.	Cross-sectional survey	PHQ-9 Categorical (0–4, 5–9, 10–14, 15–19, 20–27)	Depression was not associated with PrEP awareness or willingness to use.
Ni, Altice, Wickersham et al. 2021 [38]	Connecticut	234 individuals dependent on opioids; 51% male; 63.3% non-Hispanic White; mean age: 42.7.	Cross-sectional survey	CESD-20 (16+ screened positive)	Moderate to severe depression was not correlated with willingness to use PrEP.
Ojikutu, Bogart, Higgins-Biddle et al. 2018 [44]	United States	855 participants; 54% female; 91% Black; 4% Latino.	Cross-sectional survey	Whooley et al. 1997 2-item measure (categorical frequency)	Depressive symptoms was associated with greater PrEP willingness amongst the entire sample, but not amongst those who were PrEP indicated.
Shrestha, DiDomizio, Kim et al. 2020 [45]	New Haven, CT	234 people who use drugs; 51% male; 63% White; 79% heterosexual; mean age: 42.7.	Cross-sectional survey	CESD (16+ screened positive)	Depression was not associated with willingness to use LAI-PrEP.
Shrestha, Karki, Altice et al. 2017 [46]	New Haven, CT	400 people who use drugs; 58% male; 63% White; 15% Hispanic/Latino; 86% straight; mean age: 41.	Cross-sectional survey	CESD-20 (16+ screened positive)	Those who had moderate to severe depression were more likely to be willing to initiate PrEP.
Sullivan and Eaton 2020 [37]	Atlanta, GA	293 Black MSM; 56% gay/homosexual; mean age: 30.	Cross-sectional survey	CESD-10 (10+ screened positive)	Depression was not associated with PrEP awareness.
Tomko, Park, Allen et al. 2019 [39]	Baltimore, MD	232 female sex workers; 66% non-Hispanic White; 50% ages < 35.	Cross-sectional survey	CESD-10 (10+ screened positive)	Depressive symptoms were not associated with PrEP awareness or interest.
Willie, Kershaw, Blackstock et al. 2020 [47]	New Haven, CT; Bridgeport, CT; Hartfort, CT	271 women; 36% non-Hispanic White, 35% non-Hispanic Black; 28% Hispanic; 75% heterosexual; 61% ages 25+.	Cross-sectional survey	PHQ-9 (10+ screened positive)	Adjusting for sociodemographic variables, there was no difference between number of syndemic conditions (IPV, depression, substance use) and PrEP interest or intentions to use.

Note: PHQ = Patient Health Questionnaire, CESD = Center for Epidemiological Studies – Depression.

**Table 3:** Depression and PrEP Uptake.

Authors & Year	Location(s)	Sample Demographics	Design	Depression Measure	Depression Related Findings
Blashill, Brady, Rooney et al. 2020 [42]	San Diego, CA	151 Latino MSM men; mean age: 24.	Cross-sectional survey	PHQ-8 10+ screened positive	A psychosocial syndemic (depression, binge drinking, marijuana use, illicit polysubstance use, childhood sexual abuse) was not associated with PrEP use.
Blackstock, Platt, Golub et al. 2021 [40]	East Harlem, NY; Bronx, NY	52 women, 11.5% TGW; 34.6% non-Latina Black; 51.9% Latina/Hispanic, 42.3% LGB; mean age: 45.	Pilot test	PHQ-2 (dichotomous screening)	Depression was not associated with PrEP appointment acceptance.
Bruxvoort, Schumacher, Towner et al. 2021 [52]	Southern California	2995 PrEP candidates; 96.6% male; 60% racial/ethnic minorities; age: 50% < 35.	Retrospective Chart Review	Diagnostic codes	Depression was not associated with PrEP referral.
Eaton, Matthews, Driffin et al. 2017 [36]	Atlanta, GA; Detroit, MI; Houston, TX; Philadelphia, PA; Washington DC	1274 Black MSM and TGW; 96% male; 76% gay/same gender loving; mean age: 30.	Cross-sectional survey	CESD-10 (10+ screened positive)	In bivariate analyses, depressive symptoms were associated with PrEP use. The association did not hold in multivariable analyses.
Friedman, Sang, Bukowski et al. 2019 [34]	Atlanta, GA; Detroit, MI; Houston, TX; Memphis, TN; Philadelphia, PA; Washington DC	2398 Black MSM, 82% MSMO; 3.4% Hispanic/Latino; mean age: 29.	Serial cross-sectional survey	CESD-10 (10+ screened positive)	Depressive symptoms were not associated with PrEP use.
Krakower, Mimiaga, Rosenberger et al. 2012 [35]	United States	398 MSM 2 months prior to iPrEX publication; 82% White; 89% gay; mean age: 40. 558 MSM 2 months following publication; 84% White; 83% gay; mean age: 39.	Repeated cross-sectional survey	CESD-10 (10+ screened positive)	Depression was not associated with PrEP use.
Laborde, Kinley, Spinelli et al. 2020 [50]	San Francisco, CA	25 current or former PrEP users; 60% MSM, 20% TGW who have sex with men; 32% Black; 28% Hispanic; ages 18–57.	Qualitative Interviews	Qualitative focus	One participant reported stigma surrounding depression and HIV making it difficult to seek help.
Liu, Brown, Przybyla et al. 2021 [36]	Nashville, TN; Buffalo, NY	374 MSM, 60.2% Black; 77% gay/homosexual; median age: 26.	Cross-sectional survey	PHQ-9 (categorical: 0–4, 5–9, 10–14, 15–19, 20–27)	Depression was not associated with PrEP use.
Sullivan and Eaton 2020 [37]	Atlanta, GA	293 Black MSM; 56% gay/homosexual; mean age: 30	Cross-sectional survey	CESD-10 (10+ screened positive)	Depression alone was not associated with PrEP use. However, having two or more syndemic conditions (depression symptoms, alcohol consumption, illicit drug use, transactional sex) was associated with less likelihood of PrEP use.
Ni, Altice, Wickersham et al. 2021 [38]	Connecticut	234 individuals dependent on opioids; 51% male; 63.3% non-Hispanic White; mean age: 42.7.	Cross-sectional survey	CESD-20 (16+ screened positive)	Moderate to severe depression was not correlated with PrEP use.
Ogunbajo, Storholm, Ober et al. 2021 [48]	Southern California	30 TGW; 33.3% Black/African American; 53% Hispanic/Latina/Latinx; 46% straight/heterosexual; mean age: 30.	Mixed methods	Qualitative focus	Mental health concerns were a barrier to PrEP uptake.
Okafor, Hucks-Ortiz, Hightow-Weidman et al. 2020 [56]	Los Angeles, CA; Washington DC; Chapel Hill, NC.	226 Black MSM; 60% > 25 yrs old.	Non-randomized open-label PrEP study	CESD (10+ screened positive)	No differences were found in PrEP initiation by depression.
Serota, Rosenberg, Thorne, Sullivan, and Kelley 2019 [53]	Atlanta, GA	154 Black MSM, 45% age 22–25.	Longitudinal	PHQ-8 (10+ screened positive)	Depression was not associated with a delay between PrEP prescription and initiation.
Serota, Rosenberg, Sullivan et al. 2020 [54]	Atlanta, GA	298 non-Hispanic Black cis-gender MSM; 73% gay; 62% ages 18–21.	Prospective Observational Cohort Study	PHQ-8 (10+ screened positive)	Depressive symptoms were not associated with PrEP uptake.
Sewell, Powell, Mayer et al. 2020 [57]	United States	9697 individuals; 92% male; 69% non-Hispanic White; mean age: 43.	Cross-sectional survey	CESD (continuous)	Those who used nondaily PrEP compared with daily PrEP were more likely to have higher depressive symptoms.
Rutledge, Madden, Ogbuagu, and Meyer 2018 [51]	Connecticut	125 women involved in the criminal justice system; 69% White; mean age: 37.	Cross-sectional survey	Unspecified	There was no difference in PrEP eligibility by depressive symptoms.
Watson, Pasipanodya, Savin et al. 2020 [49]	San Diego, CA; Los Angeles, CA	37 individuals; 48% TGW, 27% trans men; 41% White, non-Hispanic; 16% Latinx/Hispanic.	Qualitative focus groups	Qualitative focus	Mental health concerns, including depression and anxiety made it difficult to access PrEP care as well as other services.
Wheeler, Fields, Beauchamp et al. 2019 [55]	Los Angeles, CA; Washington DC; Chapel Hill, NC.	226 MSM; 86% Black; 73% gay; median age: 26	Non-randomized open-label PrEP study	CESD-10 (10+ screened positive)	Depressive symptoms were not related to PrEP initiation.
Wood, Morales, Metzger et al. 2020 [58]	Philadelphia, PA	247 individuals; 89% cis male; 46% African American; 19% Latinx ethnicity; 47% ages 25–34.	Cross-sectional survey	PHQ-8 (10+ screened positive)	No associations were found between depressive symptoms and mental health treatment on PrEP use.
Carneiro, Westmoreland, Patel, and Grov 2020 [59]	United States	6283 individuals; 98% cis men; 52% White; 33% 30–39 yrs old	Cross-sectional survey	PHQ-4 (dichotomous screening)	Depression was not associated with PrEP use
Chandler, Bukowski, Matthews et al. 2020 [60]	Atlanta, GA; Detroit, MI; Houston, TX; Memphis, TN; Philadelphia, PA; Washington DC	1411 Black MSM; 80% gay/homosexual, 16% bisexual; 64% 18–29 years old	Cross-sectional survey	CESD-10 (10+ screened positive)	There was no difference between PrEP users and non-users in depressive symptoms. In considering syndemic psychosocial conditions (substance use, intimate partner violence, depression), those with greater number of syndemic conditions were more likely to report PrEP use.
Eaton, Matthews, Bukowski et al. 2018 [61]	Philadelphia, PA; Detroit, MI; Washington, DC; Atlanta, GA; Houston, TX; and Memphis, TN.	4184 Black MSM or TGW. 77.7% gay/same gender loving, mean age: 30.47.	Cross-sectional survey	CESD-10 (10+ screened positive)	Depression was associated with current PrEP use.
Moeller, Seehuus, Wahl, and Gratch 2020 [62]	United States	2406 MSM; 55% White; 18.6% Latino; 78.6% gay; mean age: 34.	Cross-sectional survey	PHQ-9 (categorical: 0–4, 5–9, 10–14, 15–19, 20+)	PrEP users reported lowest levels of depression compared with non-PrEP using participants. Use of PrEP was not associated with depression.

**Table 4:** Depression and PrEP Adherence and Retention.

Authors & Year	Location(s)	Sample Demographics	Design	Depression Measure	Depression Related Findings
Blashill, Brady, Rooney et al. 2020 [42]	San Diego, CA	151 Latino MSM; Mean age: 24.	Cross-sectional survey	PHQ-8 10+ screened positive	A psychosocial syndemic (depression, binge drinking, marijuana use, illicit polysubstance use, childhood sexual abuse) was associated with lowered self-reported adherence.
Bruxvoort, Schumacher, Towner et al. 2021 [52]	Southern California	2995 PrEP candidates; 96.6% male; 60% racial/ethnic minorities; 50% < 35 years old.	Retrospective chart review	Diagnostic codes	Depression was not associated with PrEP persistence according to medical and pharmacy records.
Colson, Franks, Wu et al. 2020 [68]	Harlem, NY	204 Black MSM (95.1%) and TGW (4.9%) newly enrolled on PrEP, 20.6% Latino; Median age: 31.	Randomized control trial	CES-D (16+ screened positive)	Those who reported depressive symptoms at baselines were less likely to be adherent according to self-report and dried blood spot (DBS) drug concentration.
Gandhi, Murnane, Bacchetti et al. 2017 [64]	Miami, Fl; San Francisco, CA; Washington DC	280 PrEP users; 99% male, 78% White; Mean age: 34.	Longitudinal	PHQ-2 (dichotomous screening)	Depressive symptoms were not associated with PrEP adherence according to DBS drug concentration.
Hoenigl, Jain, Moore 2018 [66]	Southern California	394 MSM and TGW, 99% male; 76% White; 30% Hispanic; Median age: 33.	Randomized control trial	PHQ-9 (continuous)	Baseline PHQ-9 scores were not associated with adherence according to DBS drug concentration.
Krakower, Maloney, Powell et al. 2019 [71]	Boston, MA	663 PrEP users; 96% male, 3% transgender women; 73% non-Hispanic White; Median age: 32.6.	Retrospective chart review	Medical problems list and diagnostic codes	PrEP discontinuations were more likely to occur in those who had multiple mental health disorders (includes anxiety, depression, substance use, PTSD, bipolar, schizophrenia, and attention deficit disorder).
Laborde, Kinley, Spinelli et al. 2020 [50]	San Francisco, CA	25 current or former PrEP users; 60% MSM, 20% TGW who have sex with men; 32% Black; 28% Hispanic; Ages 18–57.	Qualitative interviews	Qualitative focus	Participants indicated it was difficult to keep track of medications when having mental health problems.
Liu, Cohen, Vittinghoff et al. 2016 [65]	Miami, Fl; San Francisco, CA; Washington DC	557 PrEP users; 48% White, 34% Latino; 98% MSM; Mean age: 35.	Longitudinal	PHQ-2 (2+ screened positive)	Depression was not associated with PrEP adherence.
Mannheimer, Hirsch-Moverman, Franks et al. 2019 [67]	New York City, NY	176 MSM; 59% non-Hispanic Black; 25% Hispanic; Median age: 31.	Randomized control trial	CES-D (16+ screened positive)	Depressive symptoms were not associated with adherence measured through self-report, pill counts, and DBS drug concentration.
Ogunbajo, Storholm, Ober et al. 2021 [48]	Southern California	30 TGW; 33.3% Black/African American; 53% Hispanic/Latina/Latinx; 46% straight/heterosexual; Mean age 29.8.	Mixed methods: cross-sectional survey and qualitative interviews	Qualitative focus	Mental health concerns, including depression and anxiety, decreased motivation to take PrEP, making it less of a priority and making adherence difficult. For some, however, PrEP adherence was a coping mechanism.
Pasipanodya, Jain, Sun et al. 2018 [69]	Southern California	181 MSM; 81% White; Mean age 34.98.	Longitudinal	PHQ (categorical: 0–4, 5–9, 10–14)	Individuals with worse self-reported adherence were more likely to have depressive symptoms.
Scott, Spinelli, Vittinghoff et al. 2019 [72]	San Francisco	348 PrEP patients; 84% male; 39% White; 27% Latino; Median age: 35.	Retrospective chart review	Clinical diagnosis	Depression was not associated with discontinuations in PrEP use.
Serota, Rosenberg, Sullivan et al. 2020 [54]	Atlanta, GA	298 non-Hispanic Black cis-gender MSM; 73% gay; 62% ages 18–21.	Prospective observational cohort study	PHQ-8 (10+ screened positive)	Depressive symptoms were not associated with PrEP discontinuations.
Spinelli, Laborde, Kinley et al. 2020 [70]	San Francisco, CA	8 individuals; 63% MSM, 38% TGW who have sex with men; 38% White, 25% African American; 25% Latinx; 38% ages < 25, 38% ages 40–64.	Qualitative interviews	Qualitative focus	One participant noted depression and its intersection with substance use as a major barrier to adherence.
Wheeler, Fields, Beauchamp et al. 2019 [55]	Los Angeles, CA; Washington DC; Chapel Hill, NC.	226 MSM; 86% Black; 73% Gay; Median age: 26	Non-randomized open-label PrEP study	CESD-10 (10+ screened positive)	Depressive symptoms were not related to PrEP adherence according to DBS drug concentration.
Young, Lalley-Chareczko, Clark et al. 2020 [63]	Philadelphia, PA	31 PrEP users; 87% male, 13% TGW; 74% African American; 16% Hispanic; Mean age: 21.7.	Cross-sectional survey	PHQ-9 (10+ screened positive)	Depressive symptoms were not associated with adherence according to urine drug concentration.
Zucker, Carnevale, Richards et al. 2019 [73]	Manhattan, NY	696 PrEP users, 93% male at birth; 23% African American; 45.7% Hispanic; 54% age < 30.	Retrospective chart review	PHQ-9 (5+ screened positive)	Depressive symptoms were not associated with retention.

Note: PTSD = Post traumatic stress disorder.

**Table 5:** Effects of Anxiety across the PrEP Care Cascade.

Authors & Year	Location(s)	Sample Demographics	Design	PrEP Stage	Anxiety Measure	Anxiety Related Findings
Blackstock, Platt, Golub et al. 2021 [34]	East Harlem, NY; Bronx, NY	52 women, 11.5% TGW; 34.6% non-Latina Black; 51.9% Latina/Hispanic, 42.3% LGB; mean age: 45.	Pilot test	Awareness, initiation	GAD-2 (dichotomous screening)	Anxiety did not predict PrEP interest or appointment acceptance.
Liu, Brown, Przybyla et al. 2021 [40]	Nashville, TN; Buffalo, NY	374 MSM, 60.2% Black; 77% gay; median age: 26.	Cross-sectional survey	Awareness, initiation	GAD-7 categorical (0–4, 5–9, 10–14, 15–21)	Anxiety was not associated with PrEP awareness, willingness to use, or use.
Meyers, Rodriguez, Brill et al. 2017 [74]	New York City, NY; Philadelphia, PA	28 individuals receiving long-acting injectable cabotegravir; 60% MSM of color; 14% Latino; mean age: 31.	Mixed methods: cross-sectional survey and qualitative interviews	Uptake	Qualitative focus	Participants reported anxiety related to injections, which decreased over time and was not correlated with pain. Two forms of anxiety were present related to needles and expected pain.
Watson, Pasipanodya, Savin et al. 2020 [49]	San Diego, CA; Los Angeles, CA	37 individuals; 48% TGW, 27% trans men; 41% White, non-Hispanic; 16% Latinx/Hispanic.	Qualitative focus groups	Access	Qualitative focus	Mental health concerns, including depression and anxiety, made it difficult to access PrEP care as well as other services.
Bruxvoort, Schumacher, Towner et al. 2021 [52]	Southern California	2995 PrEP candidates; 96.6% male; 60% racial/ethnic minorities; 50% < 35 years old.	Retrospective chart review	Adherence and retention	Diagnostic codes	Anxiety was not associated with PrEP referral or persistence.
Carneiro, Westmoreland, Patel, and Grov 2020 [59]	United States	6283 individuals; 98% cis men; 52% White; 33% 30–39 years old	Cross-sectional survey	Initiation	PHQ-4 (dichotomous screening)	Anxiety was not associated with prior or current PrEP use.
Ogunbajo, Storholm, Ober et al. 2021 [48]	Southern California	30 TGW; 33.3% Black/African American; 53% Hispanic/Latina/Latinx; 46% straight/heterosexual; mean age 29.8.	Mixed methods: cross-sectional survey and qualitative interviews	Adherence and retention	Qualitative focus	Mental health concerns, including depression and anxiety, decreased motivation to take PrEP, making it less of a priority and making adherence difficult. For some, however, PrEP adherence was a coping mechanism.
Young, Lalley-Chareczko, Clark et al. 2020 [63]	Philadelphia, PA	31 PrEP users; 87% male, 13% transgender women; 74% African American; 16% Hispanic; mean age: 21.7.	Cross-sectional survey	Adherence and retention	GAD-7 (10+ screened positive)	Anxiety was associated with lower adherence according to urine drug concentration.
Scott, Spinelli, Vittinghoff et al. 2019 [72]	San Francisco	348 PrEP patients; 84% male; 39% White; 27% Latino; median age: 35.	Retrospective chart review	Adherence and retention	Diagnostic codes	Anxiety was not associated with discontinuations in PrEP use.
Krakower, Maloney, Powell et al. 2019 [71]	Boston, MA	663 PrEP users; 96% male, 3% transgender women; 73% non-Hispanic White; median age: 32.6.	Retrospective chart review	Adherence and retention	Medical problems list and diagnostic codes	PrEP discontinuations were more likely to occur in those who had multiple mental health disorders (includes anxiety, depression, substance use, PTSD, bipolar, schizophrenia, and attention deficit disorder).

Note: GAD = Generalized Anxiety Disorder scale.

**Table 6:** Effects of PrEP Use on Anxiety.

Authors & Year	Location(s)	Sample Demographics	Design	Anxiety Measure	Anxiety Related Findings
Brooks, Landovitz, Kaplan et al. 2012 [84]	Los Angeles, CA	25 gay or bisexual men in HIV serodiscordant relationships; 40% Black/African American; 32% Hispanic/Latino; 76% gay/homosexual, 24% bisexual.	Mixed methods	Qualitative focus	Having less anxiety when having sex with an HIV-positive partner was one of three reasons participants indicated for willingness to take PrEP.
Devarajan, Sales, Hunt, Comeau 2020 [77]	Atlanta, GA	10 MSM currently or formerly on PrEP; 75% Black; 95% non-Hispanic/Latino; mean Age: 30.2.	Qualitative interviews	Qualitative focus	PrEP use reduced feelings of anxiety and stress.
Hammack, Toolis, Wilson et al. 2019 [79]	New York City, NY; San Francisco, CA; Tuscon, AZ; Austin, TX	89 gay or bisexual men; 23.6% White; 24.7% Latino/Hispanic; 78.6% gay; ages 18–59.	Qualitative interviews	Qualitative focus	PrEP improved sexual culture, making sex more comfortable and enjoyable, and reducing anxiety, particularly amongst younger participants. PrEP alleviated anxiety, removing HIV-related worry.
Hojilla, Koester, Cohen et al. 2016 [75]	San Francisco, CA; Miami, FL; Washington DC	26 men on PrEP; 62% White; 27% Hispanic.	Qualitative analysis of counseling notes	Qualitative focus	PrEP alleviated anxiety related to sex and HIV, providing “peace of mind” and alleviating anxiety for both participants and their partners. As a result, some participants explored sexual roles which they otherwise would have not been comfortable with.
Mitchell, Lee, Woodyatt et al. 2016 [83]	Atlanta, GA; Detroit, MI	19 male couples, most were non-Hispanic and/or White; 28% of couples were mixed race. Specific demographics are unavailable; mean age: 33.	Qualitative interviews	Qualitative focus	Some participants reported that PrEP could be a part of couple’s agreements in order to reduce sexual anxiety and risk, to keep the couple safe.
Moeller, Seehuus, Wahl, and Gratch 2020 [62]	United States	2406 MSM; 55% White; 18.6% Latino; 78.6% gay; mean age: 34.	Cross-sectional survey	GAD-7 categorical (0–4, 5–9, 10–14, 15+)	PrEP users reported lowest levels of anxiety compared with non-PrEP-using participants. Among those on PrEP, internalized homophobia, but not number of condomless anal intercourse partners was associated with greater anxiety. In contrast, for those not on PrEP, greater number of CAI partners and internalized homophobia were associated with higher anxiety (r square = 0.06).
Mutchler, McDavitt, Ghani et al. 2015 [78]	Los Angeles, CA	24 friend pairs; 83% male; 87.5% Black; 14.6% Latino; 68.8% gay; mean age: 22.1.	Qualitative interviews	Qualitative focus	Participants described PrEP as a backup option which could provide peace of mind and reduce anxiety if other preventative methods failed.
Quinn, Christenson, Sawkin et al. 2020 [80]	Milwaukee, WI; Minneapolis, MN; Detroit, MI; and Kansas City, MO	36 Black MSM current/past PrEP users; 69% gay; mean age: 26.	Qualitative focus groups	Qualitative focus	PrEP reduced sexual and HIV-related anxiety, including relieving anxieties related to having sex, getting HIV tests, and potential fears of acquiring HIV. This reduced anxiety created sexual freedom, increasing control and autonomy regarding sexual health and providing the opportunity to explore without fears of HIV.
Storholm, Volk, Marcus et al. 2017 [81]	San Francisco, CA	30 MSM PrEP users; 40% White; 23% Latino; mean age: 27.5.	Qualitative interviews	Qualitative focus	Individuals indicated PrEP enhanced their sexual wellbeing, decreasing HIV related anxiety, and increasing openness to HIV positive partners.
Whitfield, Jones, Wachman et al. 2021 [85]	United States	1071 cis MSM PrEP users; 70% White; 11% Latino; 38.7% ages 25–34.	Longitudinal	Sexual anxiety MSSCQ subscale (continuous)	Participants showed less sexual anxiety when on PrEP compared to their sexual anxiety before beginning PrEP.
Yang, Krishnan, Kelley et al. 2020 [82]	Baltimore, MD	18 MSM PrEP users; 83% non-Hispanic Black/African American.	Qualitative interviews	Qualitative focus	Participants reported PrEP as providing “peace of mind”, reducing anxiety related to HIV or partner dishonesty.
Zapata, Petroll, de St. Aubin, and Quinn 2020 [76]	Milwaukee, WI	20 PrEP patients; 93% cis male; 60% White; 20% Hispanic; 85% gay; mean age: 33.	Qualitative interviews	Qualitative focus	PrEP helped alleviate HIV-related anxiety, allowing individuals to feel they were being responsible.

Note: MSSCQ = Multidimensional Sexual Self Concept Questionnaire.

## Data Availability

Not applicable.
